# Effects of environmental factors on genetic diversity of *Caragana microphylla* in Horqin Sandy Land, northeast China

**DOI:** 10.1002/ece3.2549

**Published:** 2016-10-20

**Authors:** Wenda Huang, Xueyong Zhao, Xin Zhao, Yulin Li, Jie Lian

**Affiliations:** ^1^ Northwest Institute of Eco‐Environment and ResourcesChinese Academy of SciencesLanzhouChina

**Keywords:** *Caragana microphylla*, environmental factors, genetic diversity, Horqin Sandy Land, intersimple sequence repeat

## Abstract

*Caragana microphylla* (Leguminosae) is a dominant climax semishrub species in northern China. We evaluated genetic variation within and among populations sampled from three different environmental gradients in Horqin Sandy Land in northern China using intersimple sequence repeats markers and investigated the possible existence of relationships between genetic diversity and environmental factors. The results showed that *C. microphylla* have high genetic diversity, and environmental gradients affected genetic diversity of *C. microphylla* populations. Genetic diversity of all populations was affected by many environmental factors and as well correlated with warm index and soil Olsen phosphorus (SOP) concentration. These results have important implications for restoration and management of these degraded ecosystems in arid and semi‐arid areas.

## 1 Introduction

The environment plays an important role in evolutionary processes (Scheiner, 1993). A recent surge of studies on plants has explicitly analyzed trait variation among and within species along environmental gradients (Hulshof et al., 2013; Read, Moorhead, Swenson, Bailey, & Sander, 2014). Changes in environmental conditions are predicted to alter diversity within populations (Lovejoy & Hannah, 2005), and genetic diversity is thought to play an essential role in the survival of plant populations in dynamic environments. Environmental factors affect the dynamics of species, even those with high potential for gene flow (Sork et al., 2010; Freeland, Biss, Conrad, & Silvertown, 2010); in turn, genetic diversity of individuals within a population can also affect a range of ecological factors (Vellend & Genber, 2005). The interaction between genetic diversity and ecological factors has been assessed in a few population‐level studies in plants (Hughes, Inouye, Johnson, Underwood, & Vellend, 2008). Genetic variation among plant populations often occurs along different climatic gradients, such as temperature and precipitation gradients (Keller et al., 2011). For example, several studies demonstrated significant effects of simulated climate change on species composition and genetic structure in temperate grassland ecosystems (Fridley, Grime, Askew, Moser, & Stevens, 2011; Harte & Shaw, 1995; Wu, Dijkstra, Koch, & Hungate, 2012).

Genetic variation is nonrandomly distributed among populations and species (Nevo, 1998), with distribution of alleles and genotypes over space and time often affected by numerous factors such as breeding system, seed dormancy and dispersal mechanism, geographic variation and range, life span and other life‐history traits, natural selection, and the history of populations (population genetics, phylogeography, and landscape ecology). (Faye et al., 2016; Hamrick & Godt, 1989; Hamrick, Godt, Murawski, & Loveless, 1991; Hanin, Quaye, Westberg, & Barazani, 2013; Maki, 2003; Manel, Schwartz, Luikart, & Taberlet, 2003; Meloni, Perini, Filigheddu, & Binelli, 2006; Su & Zhang, 2014). Populations in different parts of a species’ range and microhabitats experience and respond to climate change differently (Rehfeldt, Crookston, Warwell, & Evans, 2006; Rehfeldt et al., 2002). This differential response is because of both the genetic composition of local populations and magnitude of climate change, which also vary geographically (Sork et al., 2010). Environmental factors are often responsible for the patterns of genetic structure observed at small spatial scales (Sacks, Brown, & Ernest, 2004). Range expansions characterized by short‐distance dispersal result in reduced genetic diversity in populations at the expanding range front, because these populations suffer from sequential founding events and genetic drift (Austerlitz, Jung, Godelle, & Gouyon, 1997). Moreover, projections of increased temperatures, more frequent droughts, habitat fragmentation, and declining population size indicate that it is questionable whether many plant species will be able persist in their current distributions under such conditions (Richter et al., 2012). Environmental tolerance or local adaptation might allow some species to persist in parts of their current range; other species, even if currently protected, will survive only by colonizing newly suitable areas (Araújo, Cabeza, Thuiller, Hannah, & Williams, 2004).

Horqin Sandy Land is located in the agropastoral transitional zone between the Inner Mongolian Plateau and the Northeast Plains (42°41′–45°45′N, 118°35′–123°30′E) and is one of the four largest sandy areas in northern China; it covers an area of approximately 139,300 km^2^, which had been desertified sandy land area up to 71,884 km^2^ of which is desertified sandy land (Wang, 2003; Zhao, Zhao, & Zhang, 2003). Landscape in this area is characterized by sand dunes that alternate with gently undulating lowland areas (Li, Zhang, Duan, & Kang, 2005). This area belongs to the continental semi‐arid monsoon climate and is in the temperate zone, with a mean annual temperature (AMT) of 3–7°C and mean annual rainfall (AP) of 350–500 mm (Zhao et al., 2003). Over recent decades, this region has undergone severe desertification (Li, Zhao, Zhang, Zhang, & Shirato, 2004; Li et al., 2000) and has displayed the northern moving phenomenon of the interlocked agropasturing area of north China in the most recent hundred years (Zhao, Zhao, & Zhang, 2000; Zhao, Zhao, Zhang, & Zhou, 2002).

*Caragana microphylla* (Leguminosae) is a climax and dominant sand‐fixing shrub species native to northern China; it is an important component of vegetation rehabilitation efforts in the northern China, because it has several highly valuable ecological traits, which include high drought tolerance, antiwind erosion properties, and N_2_‐fixation capacity (Han, Wang, & Gao, 2011; Zhang et al., 2009; Zhao, 2005), and it is widely planted throughout severely desertified sites to control land desertification in northern China (Su, Zhang, Li, & Wang, 2005; Zhang, Su, Cui, Zhang, & Chang, 2006). *Caragana microphylla* is distributed in semifixed and fixed sand dunes, and Horqin Sandy Land is the main distribution regions. A unique set of conditions with respect to precipitation and temperature are found in northern China, and Inner Mongolia characterizes the main portion of this species’ distribution (Fu, 1993). *Caragana microphylla* is long‐lived, perennial, insect‐pollinated, has seed‐based reproduction, and has a broad ecological amplitude (Fu, 1993). Previous studies on *C. microphylla* have focused on aspects of population distribution patterns and ecological adaptations (Zhao, 2005), morphological characteristics and variations (Li, Jiang, Gu, Ma, & Gao, 2008), physiological adaptations (Li et al., 2008; Ma, Gao, Liu, Wang, & Guo, 2003), nutrient absorption (Li, Chen, Cui, Zhao, & Zhang, 2013), and genetic diversity (Guo et al., 2008; Huang, Zhao, Zhao, Li, & Pan, 2016; Huang et al., 2013). However, the relationship between *C. microphylla* genetic diversity and environmental gradients has not yet been reported.

The association between genetic and environmental gradients is well‐established evidence of natural selection (Endler, 1986; Manel et al., 2010). In this study, we assessed *C. microphylla* population genetic variation in different environmental gradients in Horqin Sandy Land using intersimple sequence repeat (ISSR) markers. We used the canonical approach to investigate the potential association between genetic variation and environment, based on correlation analysis between a measure of genetic diversity and environmental variables (Vasemägi & Primmer, 2005).

The aim was to find out how to respond to environmental conditions change in genetic diversity of *C. microphylla*.

1. Is there a difference in genetic diversity of *C. microphylla* along with environmental gradients change (habitat, temperature, and humidity gradients)? If this is the case, what is the change trend and characteristics of genetic diversity within and among *C. microphylla* populations along environmental gradients across Horqin Sandy Land.
2. Is there correlation between genetic diversity of *C. microphylla* and climatic factors in Horqin Sandy Land? If this is the case, whether consistent with previous findings (AP and CI were the major climatic factors that affected genetic diversity indices of *C. microphylla* populations from northern China; Huang et al., 2016)? If not consistent, which one or several climatic factors affect genetic diversity of *C. microphylla* populations from Horqin Sandy Land, and how are they correlated?
3. Is there correlation between genetic diversity of *C. microphylla* and soil factors in Horqin Sandy Land? If this is the case, which one or several soil factors affect genetic diversity of *C. microphylla* populations from Horqin Sandy Land, and how are they correlated?

In this article, we explore these hypotheses using ISSR markers and environmental data and provide more information on the genetic diversity of *C. microphylla* in Horqin Sandy Land, which might be applicable in restoration and management of degraded ecosystems in arid and semi‐arid regions.

## 2 Materials and Methods

### 2.1 Sampling

**Figure 1:**
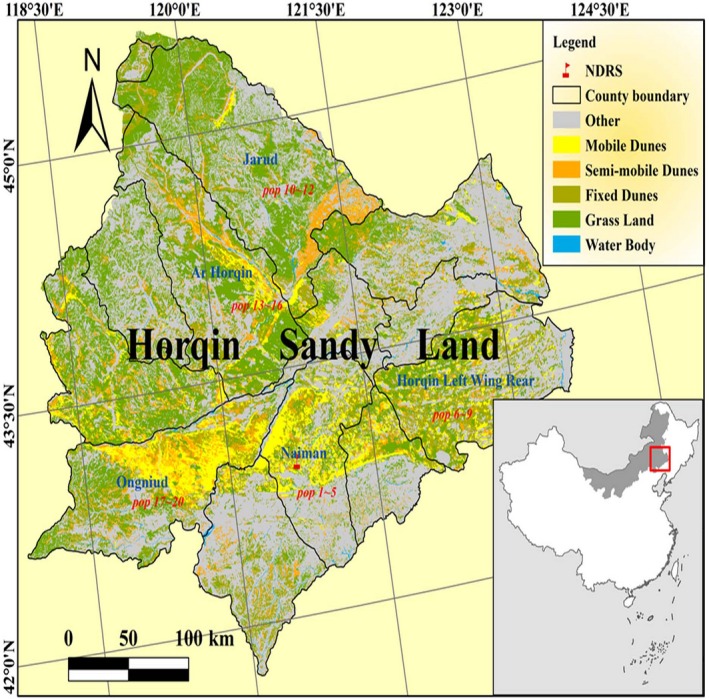
Sampling sites of 20 populations of *Caragana microphylla* in Horqin Sandy Land

A total of 260 individuals were sampled from 20 natural *C. microphylla* populations. We sampled 13 individuals from each population (Table 1, Figure 1). Climatic data were obtained from the China Meteorological Administration and are shown in Table 2; climatic means were derived from data from 1971 to 2000. Annual temperature range (ART) was calculated from the formula,

ART=(MTWM−MTCM)

where MTWM* *= warmest monthly mean temperature, MTCM* *= coldest monthly mean temperature. Warm index (*WI*) values were calculated from the formula,

$$WI=\sum_{i=1}^{12}\left(t_{i}-5\right)$$

where *t *= greater than 5°C monthly mean temperature. Cold index (*CI*) values were calculated from the formula,

$$CI=\sum_{i=1}^{12}\left(5-t_{i}\right)$$

where *t *= lower than 5°C monthly mean temperature. Hydrothermal synthesis index (*S*) values were calculated from the formula,

$$S=\sum_{t=1}^{12}0.18r_{t}/1.045^{T_{t}}$$

where *t *= month, *r*_*t*_ = monthly rainfall, and *T*_*t*_ = monthly mean temperature (Bailey, 1979).

**Table 1:** Origin of materials and number of samples for 20 populations of *Caragana microphylla* from Horqin sandy land

Population	No of plants	Latitude (°N)	Longitude (°E)	Altitude (m)	Habitats
Pop1	13	43°10′10″	120°37′50″	434	Mobile sand dune
Pop2	13	42°57′35″	120°40′46″	357	Lowlands between mobile sand dunes
Pop3	13	42°55′46″	120°41′38″	367	Fixed sand dune
Pop4	13	42°55′45″	120°41′37″	350	Lowlands between fixed sand dunes
Pop5	13	43°26′48″	120°01′26″	368	Semifixed sand dune
Pop6	13	43°04′03″	122°17′31″	239	Mobile sand dune
Pop7	13	43°04′03″	122°17′31″	239	Lowlands between mobile sand dunes
Pop8	13	43°08′34″	122°14′47″	220	Fixed sand dune
Pop9	13	43°08′34″	122°14′47″	220	Lowlands between fixed sand dunes
Pop10	13	44°00′09″	121°57′15″	186	Mobile sand dune
Pop11	13	44°00′09″	121°57′15″	186	Lowlands between mobile sand dunes
Pop12	13	44°25′33″	121°16′02″	226	Lowlands between fixed sand dunes
Pop13	13	44°13′21″	120°22′48″	362	Mobile sand dune
Pop14	13	44°13′21″	120°22′48″	362	Lowlands between mobile sand dunes
Pop15	13	43°51′40″	120°13′46″	437	Fixed sand dune
Pop16	13	43°40′34″	120°28′26″	325	Semifixed sand dune
Pop17	13	43°22′26″	119°33′02″	442	Mobile sand dune
Pop18	13	43°22′26″	119°33′02″	442	Lowlands between mobile sand dunes
Pop19	13	43°05′46″	119°36′48″	485	Lowlands between fixed sand dunes
Pop20	13	43°09′59″	119°34′33″	477	Semifixed sand dune

**Table 2:** Climatic factors value (average ± *SD*) for the 20 population sites from the Horqin Sandy Land, obtained from China Meteorological Administration

Population	Location	AMT (±*SD*) (°C)	ART (±*SD*) (°C)	*WI* (±*SD*) (°C)	*CI* (±*SD*) (°C)	AP (±*SD*) (mm)	*S* (±*SD*)
Pop1‐Pop5	Naiman	7.5 ± 0.4	36.1 ± 1.8	56.3 ± 6.7	86.2 ± 0.3	269.9 ± 40.2	21.8 ± 1.9
Pop6‐Pop9	Horqin Left Wing Rear	6.9 ± 0.6	38.5 ± 2.3	60.4 ± 7.2	80.4 ± 2.8	448.1 ± 39.7	36.1 ± 0.5
Pop10‐Pop12	Jarnd	6.6 ± 0.5	36.5 ± 2.3	61.2 ± 4.2	80.2 ± 8.5	382.5 ± 145.9	29.8 ± 9.7
Pop13‐Pop16	Ar Horqin	5.9 ± 0.8	35.9 ± 2.0	74.7 ± 6.4	64.0 ± 4.7	369.7 ± 121.6	30.2 ± 9.9
Pop17‐Pop20	Ongniud	6.4 ± 0.5	34.7 ± 4.5	58.7 ± 3.7	75.7 ± 6.6	369.9 ± 102.3	31.3 ± 9.1

AMT, mean annual temperature; ART, annual temperature range; *WI*, warm index; *CI*, cold index; AP, mean annual rainfall; *S*, hydrothermal synthesis index.

This study evaluated three environmental gradients. (1) Habitat gradients: mobile sand dune (vegetation coverage <10%), fixed sand dune (vegetation coverage >50%), semifixed sand dune (vegetation coverage = 30–50%), and lowlands between sand dunes. (2) Thermodynamic gradients: low temperature region (AMT <6.0°C), middle temperature (AMT = 6.0–7.0°C), and high temperature (AMT >7.0°C). (3) Humidity gradients: low humidity (AP <300 mm), middle humidity (AP = 300–400 mm), high humidity region (AP >400 mm).

At each location, a composite soil sample from three depths (0–10, 10–20, and 20–30 cm) was collected from nine sampling points. Soil samples were air‐dried and hand‐sieved through a 2‐mm screen to remove roots and other debris. A portion of each air‐dried soil sample was ground to pass a 0.1‐mm mesh for soil nutrient analyses (Table 3). Soil organic carbon (SOC) concentration was determined using the Walkley–Black dichromate oxidation procedure (Nelson & Sommers, 1982). Soil available nitrogen (SAN) was determined using the alkaline diffusion method, and soil Olsen phosphorus (SOP) was determined using the Olsen method (Olsen, Cole, Watanabe, & Dean, 1954). Young healthy leaves were arbitrarily sampled from plants spaced at least 30 m apart and immediately stored with silica gel in ziplock plastic bags for later DNA extraction.

**Table 3:** Soil factors value (average ± *SD*) for the 20 population sites from the Horqin Sandy Land

Population	SOC (±*SD*) (g/kg)	SAN (±*SD*) (g/kg)	SOP (±*SD*) (g/kg)	SOC/SAN (±*SD*)	SOC/SOP (±*SD*)	SAN/SOP (±*SD*)
Pop1	0.118 ± 0.019	0.004 ± 0.001	0.006 ± 0.000	31.837 ± 8.100	18.624 ± 2.833	0.603 ± 0.990
Pop2	0.113 ± 0.024	0.004 ± 0.002	0.003 ± 0.000	29.021 ± 10.003	46.094 ± 12.005	1.732 ± 0.413
Pop3	1.870 ± 0.822	0.008 ± 0.002	0.006 ± 0.002	248.033 ± 88.055	30.805 ± 125.667	1.267 ± 0.307
Pop4	2.495 ± 0.773	0.007 ± 0.003	0.006 ± 0.002	392.014 ± 101.067	419.009 ± 34.012	1.115 ± 0.236
Pop5	0.360 ± 0.099	0.004 ± 0.001	0.004 ± 0.001	100.002 ± 26.953	90.055 ± 45.333	0.870 ± 0.303
Pop6	0.867 ± 0.180	0.018 ± 0.003	0.015 ± 0.008	48.095 ± 5.701	94.160 ± 91.307	2.014 ± 1.884
Pop7	1.336 ± 0.696	0.023 ± 0.006	0.010 ± 0.003	54.002 ± 15.333	16.258 ± 7.126	3.049 ± 2.495
Pop8	1.930 ± 0.990	0.027 ± 0.010	0.026 ± 0.014	70.043 ± 10.015	180.077 ± 72.225	2.600 ± 3.310
Pop9	1.920 ± 0.840	0.028 ± 0.007	0.007 ± 0.003	70.051 ± 10.005	360.089 ± 100.250	5.480 ± 3.290
Pop10	1.204 ± 0.351	0.021 ± 0.004	0.006 ± 0.004	56.400 ± 13.729	288.432 ± 72.025	4.953 ± 2.781
Pop11	0.300 ± 0.093	0.009 ± 0.002	0.004 ± 0.001	34.067 ± 7.046	95.123 ± 35.467	2.966 ± 1.736
Pop12	1.860 ± 0.790	0.022 ± 0.005	0.008 ± 0.004	80.095 ± 20.028	260.065 ± 112.678	3.670 ± 3.300
Pop13	0.474 ± 0.186	0.012 ± 0.002	0.007 ± 0.000	39.918 ± 15.805	73.663 ± 33.111	1.828 ± 0.225
Pop14	0.692 ± 0.617	0.013 ± 0.006	0.007 ± 0.001	49.006 ± 16.002	133.004 ± 45.161	2.296 ± 1.682
Pop15	1.380 ± 0.420	0.019 ± 0.004	0.006 ± 0.003	70.046 ± 10.051	350.089 ± 102.233	4.990 ± 3.420
Pop16	2.306 ± 1.355	0.026 ± 0.008	0.025 ± 0.007	100.013 ± 30.105	100.047 ± 43.915	1.100 ± 0.400
Pop17	1.176 ± 1.298	0.017 ± 0.010	0.010 ± 0.004	58.291 ± 23.838	265.794 ± 57.881	3.101 ± 4.876
Pop18	0.598 ± 0.330	0.014 ± 4.7700	0.007 ± 0.002	41.037 ± 10.099	78.095 ± 30.009	1.917 ± 0.410
Pop19	2.110 ± 1.610	0.025 ± 12.7100	0.009 ± 0.005	80.095 ± 20.012	230.75 ± 80.078	2.870 ± 0.770
Pop20	3.128 ± 1.403	0.040 ± 13.9156	0.031 ± 0.001	78.205 ± 14.333	100.903 ± 45.533	1.290 ± 0.473

SOC, soil organic carbon; SAN, soil available nitrogen; SOP, soil Olsen phosphorus.

### 2.2 DNA extraction and ISSR‐PCR amplification

Total DNA was extracted using an AxyPrep genomic DNA mini kit (Axygen, Beijing, China). DNA was quantified spectrophotometrically; samples that yielded high quantities of good‐quality DNA were used in consecutive experiments. After screening 100 ISSR primers from the University of British Columbia (UBC primer set no. 9) for well‐amplified and polymorphic bands among plant populations, we selected 15 primers for use with all individuals.

ISSR amplifications were performed in 25‐μl reaction volumes that contained 40 ng genomic DNA, 1.0 U Taq polymerase, 3 mmol/L MgCl_2_, 500 μmol/L of each dNTP, 20 mmol/L Tris–HCl (pH 8.3), 100 mmol/L KCl, and 0.3 μmol/L primer. Amplification conditions consisted of an initial step of 3 min at 94°C, followed by 35 cycles of 45 s at 94°C, 45 s at the appropriate annealing temperature (see Table 4 for details), and 2 min at 72°C, and a final 7‐min extension step at 72°C. ISSR reactions were performed at least twice for all individuals and primers to determine the reproducibility of banding patterns. Amplification products along with a 100‐bp DNA ladder were electrophoretically resolved on 1.8% agarose gels that contained ethidium bromide (0.5 μg/ml final concentration) at 100 V for 2 hr and were photographed under ultraviolet light.

**Table 4:** Primers sequence, melting temperature and percentage of polymorphism in ISSR analyses of *Caragana microphylla*

Primers	Sequences (5′ → 3′)	*T*_m_ (°C)	No. bands	No. polymorphic loci	Percentage of polymorphic loci	Amplified band size (bp)
UBC 807	(AG)_8_T	54	25	25	100.00	150–1750
UBC 810	(GA)_8_T	53	19	16	84.21	150–1500
UBC 826	(AC)_8_C	54	18	17	94.44	250–1250
UBC 827	(AC)_8_G	54	14	14	100.00	250–2000
UBC 835	(AG)_8_YC	53	16	14	87.50	150–1250
UBC 836	(AG)_8_YA	52	20	19	95.00	150–1350
UBC 840	(GA)_8_YT	58	15	13	86.67	200–1500
UBC 842	(GA)_8_YG	58	27	25	92.59	150–1500
UBC 848	(CA)_8_RG	48	13	13	100.00	225–1250
UBC 857	(AC)_8_YG	58	26	24	92.31	150–1750
UBC 864	(ATG)_6_	52	23	23	100.00	250–2000
UBC 880	(GGAGA)_3_	58	25	24	96.00	100–2000
UBC 889	DBD(AC)_7_	52	14	13	92.86	100–900
UBC 892	TAGATCTGATATCTGAATTCCC	48	18	16	88.89	500–2000
UBC 895	AGAGTTGGTAGCTCTTGATC	48	15	13	86.67	300–2000

*y* = *c*/*t*;* b* = *c*/*g*/*t*;* d* = *a*/*g*/*t*.

### 2.3 Data analysis

During analysis of the resulting gels, only clear and reproducible bands were considered. Amplified fragments were scored for the presence (1) or absence (0) of bands, and the data transformed into a 0/1 binary character matrix. The resulting binary data matrix was analyzed using POPGENE version 1.32 (Yeh & Yang, 1999). Genetic diversity of each population was estimated according to percentage of polymorphic loci (*P*), observed number of alleles (*N*_a_), effective number of alleles (*N*_e_), Nei's genetic diversity (*h*), Shannon's diversity index (*I*), gene differentiation coefficient (*G*_st_), and gene flow (*N*_m_). Redundancy analysis (RDA) was used to determine the relative contribution of the measured environmental variables to genetic diversity indices of *C. microphylla*. Data were first analyzed by detrended correspondence analysis, which indicated that RDA was an appropriate approach (gradient length <3). To avoid overfitting in the regression model due to the large number of explanatory variables, the most discriminating variables were selected using the “forward selection” procedure of the program during analysis. Genetic diversity and environmental data were log (*x* + 1)‐transformed prior to analysis. RDA was performed using CANOCO version 4.5 (ter Braak & Šmilauer, 2002).

## 3 Results

### 3.1 ISSR profiles and genetic diversity

Intersimple sequence repeat band profiles revealed high levels of polymorphism in the surveyed *C. microphylla* populations. The 15 selected ISSR primers generated a total of 288 clear and distinguishable fragment bands, of which 269 (93.40%) were polymorphic. The size of the amplified fragments ranged from 100 to 2,000 bp, with 19.2 fragments generated on average per primer. The greatest number of bands was generated with the primer UBC842. The least number of bands was generated from the primer UBC848. The percentage of polymorphic bands ranged from 84.21% to 100%. The highest percentage of polymorphic bands was generated with the primers UBC807, UBC827, UBC848, and UBC864. The lowest percentage of polymorphic bands was generated with the primer UBC810 (Table 4).

Calculated genetic diversities within and among the 20 *C. microphylla* populations are given in Table 5. The number of polymorphic loci (*n*) ranged from 19 to 121, and *P* ranged from 6.60% to 42.01%. *N*_a_ varied from 1.0660 to 1.4201; *N*_e_ ranged from 1.0385 to 1.3116. *h* and *I* values were 0.0233–0.1702 and 0.0352–0.2486, respectively. The highest genetic diversity indices were in population 15, and the lowest genetic diversity values were in population 4. At the species level, *h* was 0.3365, and *I* was 0.5041 (Table 5). AMOVA showed that most of the variation (>88.94%) was within populations (Table 6).

**Table 5:** Genetic diversity indices of *Caragana microphylla* populations

Populations	Sample size	*n*	*P* (%)	*N*_a_	*N*_e_	*h*	*I*
Pop1	13	50	17.36	1.1736 ± 0.3794	1.1190 ± 0.2793	0.0683 ± 0.1541	0.1004 ± 0.2236
Pop2	13	29	10.07	1.1007 ± 0.3014	1.0661 ± 0.2135	0.0385 ± 0.1189	0.0570 ± 0.1737
Pop3	13	27	9.38	1.0938 ± 0.2920	1.0649 ± 0.2156	0.0371 ± 0.1191	0.0544 ± 0.1726
Pop4	13	19	6.60	1.0660 ± 0.2487	1.0385 ± 0.1580	0.0233 ± 0.0916	0.0352 ± 0.1358
Pop5	13	32	11.11	1.1111 ± 0.3148	1.0720 ± 0.2249	0.0417 ± 0.1233	0.0619 ± 0.1797
Pop6	13	40	13.89	1.1389 ± 0.3464	1.0965 ± 0.2579	0.0549 ± 0.1420	0.0804 ± 0.2052
Pop7	13	54	18.75	1.1875 ± 0.3910	1.1283 ± 0.2857	0.0740 ± 0.1586	0.1089 ± 0.2306
Pop8	13	113	39.24	1.3924 ± 0.4891	1.3116 ± 0.4107	0.1700 ± 0.2177	0.2441 ± 0.3092
Pop9	13	55	19.10	1.1910 ± 0.3938	1.1188 ± 0.2608	0.0714 ± 0.1510	0.1066 ± 0.2230
Pop10	13	65	22.57	1.2257 ± 0.4188	1.1376 ± 0.2811	0.0820 ± 0.1596	0.1228 ± 0.2344
Pop11	13	52	18.06	1.1806 ± 0.3853	1.1227 ± 0.2853	0.0701 ± 0.1560	0.1032 ± 0.2257
Pop12	13	37	12.85	1.1285 ± 0.3352	1.0838 ± 0.2395	0.0484 ± 0.1323	0.0717 ± 0.1924
Pop13	13	59	20.49	1.2049 ± 0.4043	1.1322 ± 0.2875	0.0768 ± 0.1591	0.1140 ± 0.2317
Pop14	13	58	20.14	1.2014 ± 0.4017	1.1346 ± 0.2914	0.0777 ± 0.1613	0.1146 ± 0.2342
Pop15	13	121	42.01	1.4201 ± 0.4944	1.2992 ± 0.3802	0.1702 ± 0.2076	0.2486 ± 0.2988
Pop16	13	104	36.11	1.3611 ± 0.4812	1.2794 ± 0.3985	0.1532 ± 0.2113	0.2208 ± 0.3005
Pop17	13	51	17.71	1.1771 ± 0.3824	1.1135 ± 0.2679	0.0664 ± 0.1495	0.0987 ± 0.2183
Pop18	13	32	11.11	1.1111 ± 0.3148	1.0870 ± 0.2505	0.0483 ± 0.1382	0.0694 ± 0.1980
Pop19	13	38	13.19	1.1319 ± 0.3390	1.0832 ± 0.2278	0.0496 ± 0.1309	0.0738 ± 0.1928
Pop20	13	90	31.25	1.3125 ± 0.4643	1.2193 ± 0.3567	0.1242 ± 0.1926	0.1819 ± 0.2771
Average	13	56.3	19.55	1.1955 ± 0.3789	1.1354 ± 0.2786	0.0773 ± 0.1537	0.1134 ± 0.2229
Species level	260	288	93.40	1.9931 ± 0.0832	1.5758 ± 0.3162	0.3365 ± 0.1470	0.5041 ± 0.1847

*n*, the number of polymorphic loci; *P*, the percentage of polymorphic loci; *N*_a_, observed number of alleles; *N*_e_, effective number of alleles; *h*, Nei's gene diversity; *I*, Shannon's information index.

**Table 6:** Analysis of molecular variance (AMOVA) for *Caragana microphylla* populations

Source of variation	*df*	Percentage of variation	Fixation indices	*p*
Among populations^a^	19	11.06	*F*_ST_ = 0.1106	<.001
Within population	268	88.94		
Total	287			
Among groups^b^	2	8.71	*F*_CT_ = 0.0871	.005
Among populations with groups	1	1.80	*F*_SC_ = 0.0928	<.001
Total	268	89.50	*F*_ST_ = 0.1055	<.001
Among groups^c^	2	8.65	*F*_CT_ = 0.0865	.004
Among populations with groups	1	1.83	*F*_SC_ = 0.0937	<.001
Total	268	89.52	*F*_ST_ = 0.1072	<.001

a AMOVA from 20 populations as one group.

b AMOVA from three groups as represented by *WI*.

c AMOVA from three groups as represented by SOP.

### 3.2 Pattern of population genetic and its correlation with environmental gradients

Genetic diversity of *C. microphylla* populations from different environmental gradients is given in Table 7. Along the habitat gradients, *N*_a_ varied from 1.1294 to 1.3021, *N*_e_ ranged from 1.0811 to 1.2252, *h* ranged from 0.0482 to 0.1258, and *I* values ranged from 0.0718 to 0.1824; *G*_st_ ranged from 0.5017 to 0.8384, and *N*_m_ ranged from 0.0964 to 0.4967. Compared with other habitats, lowland populations between fixed sand dunes had the greatest genetic diversity, and those in semifixed sand dunes had the least genetic diversity. Along thermodynamic gradient, *N*_a_ varied from 1.1090 to 1.2969, *N*_e_ ranged from 1.0721 to 1.2114, *h* ranged from 0.0418 to 0.1195, *I* ranged from 0.0618 to 0.1745, *G*_st_ ranged from 0.5663 to 0.6864, and *N*_m_ ranged from 0.2284 to 0.3830. The greatest genetic diversity was in populations in medium‐temperature region populations, and the least genetic diversity was in populations in high‐temperature regions. Along the humidity gradient, *C. microphylla* populations from high‐humidity regions had the greatest genetic diversity, and low‐humidity regions had the least genetic diversity: *N*_a_ varied from 1.1090 to 1.2275, *N*_e_ ranged from 1.0721 to 1.1638, *h* ranged from 0.0418 to 0.0926, *I* ranged from 0.0618 to 0.1350; *G*_st_ ranged from 0.5961 to 0.6864, and *N*_m_ ranged from 0.2284 to 0.3388.

**Table 7:** Genetic diversity indices of *Caragana microphylla* populations from environmental gradients

Environmental gradients		*N*_a_	*N*_e_	*h*	*I*	*G*_st_	*N*_m_
Habitat gradients	Mobile sand dune	1.1840 ± 0.0330	1.1198 ± 0.0162	0.0697 ± 0.0104	0.1032 ± 0.0162	0.7609	0.1571
	Lowlands between mobile sand dunes	1.1563 ± 0.0467	1.1077 ± 0.0297	0.0617 ± 0.0173	0.0906 ± 0.0257	0.7980	0.1266
	Fixed sand dune	1.2616 ± 0.1326	1.1902 ± 0.1067	0.1064 ± 0.0579	0.1549 ± 0.0828	0.5731	0.3725
	Lowlands between fixed sand dunes	1.3021 ± 0.1809	1.2252 ± 0.1390	0.1258 ± 0.0768	0.1824 ± 0.1108	0.5017	0.4967
	Semifixed sand dune	1.1294 ± 0.0511	1.0811 ± 0.0330	0.0482 ± 0.0197	0.0718 ± 0.0292	0.8384	0.0964
Thermodynamic gradients	Low‐temperature region	1.1783 ± 0.0486	1.1147 ± 0.0278	0.0669 ± 0.0170	0.0992 ± 0.0258	0.6223	0.3035
	Medium‐temperature region	1.2969 ± 0.1109	1.2114 ± 0.0904	0.1195 ± 0.0493	0.1745 ± 0.0704	0.5663	0.3830
	High‐temperature region	1.1090 ± 0.0397	1.0721 ± 0.0292	0.0418 ± 0.0164	0.0618 ± 0.0239	0.6864	0.2284
Humidity gradients	Low‐humidity region	1.1090 ± 0.0397	1.0721 ± 0.0292	0.0418 ± 0.0164	0.0618 ± 0.0239	0.6864	0.2284
	Medium‐humidity region	1.1832 ± 0.0905	1.1258 ± 0.0638	0.0721 ± 0.0357	0.1060 ± 0.0523	0.6862	0.2286
	High‐humidity region	1.2275 ± 0.1125	1.1638 ± 0.0994	0.0926 ± 0.0523	0.1350 ± 0.0739	0.5961	0.3388

### 3.3 Correlation between climatic variation and genetic variation

**Figure 2:**
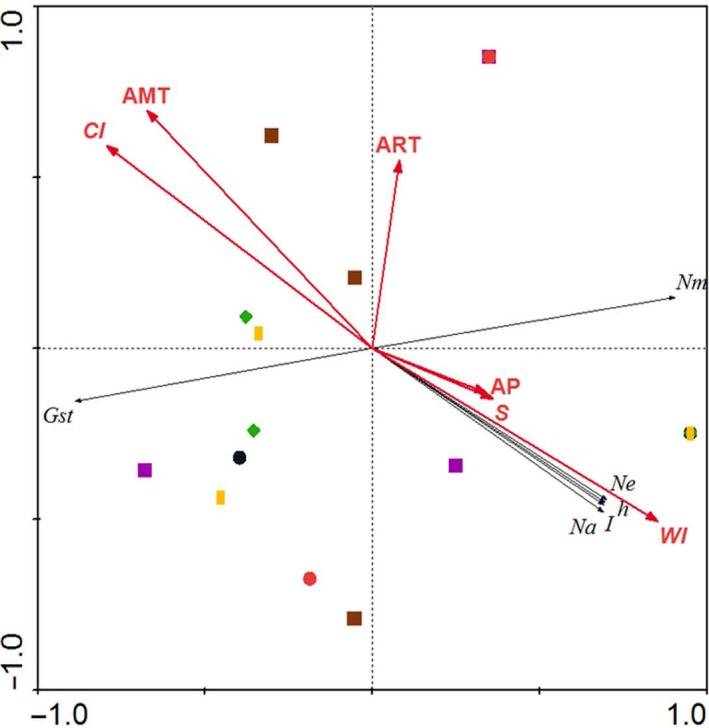
Correlation coefficients between genetic diversity of 20 *Caragana microphylla* populations and climatic factors

Genetic diversity of the 20 *C. microphylla* populations from Horqin Sandy Land was influenced by the following climatic factors: AMT, ART, *WI*,* CI*, AP, and *S* (Figure 2). Correlation analyses evidenced that there were positive (*WI*, AP, and *S*) and negative (AMT and *CI*) correlations between genetic diversity indices of *C. microphylla* populations and climatic factors (Figure 2). RDA showed that the six climate environmental variables (AMT, ART, AP, *CI*,* WI*, and *S*) together explained 78.4% of the total variation in the data, with axes 1 and 2 explaining 69.3% and 9.1% of the total variation, respectively (Figure 2). Of the six environmental variables, only *WI* was significant according to the Monte Carlo permutation test (*p* = .001), whereas those of the remaining variables were not significant according to the Monte Carlo permutation test (in all cases *p* > .05). Of the 78.4% total variation explained by RDA, 57.1% was explained by *WI*, and the rest (21.3%) by variables that were not significant according to the Monte Carlo permutation test.

Using RDA, we assessed the relative contribution of the measured climate environmental factors in determining genetic diversity of *C. microphylla* populations. RDA revealed that the six environmental factors (AMT, ART, AP, *CI*,* WI*, and *S*) together explained 78.4% of the total variation in the genetic diversity indices of *C. microphylla* populations from Horqin Sandy Land. This result indicates that some other climate factors that were not considered in this study also contribute to the unexplained variation, and *WI* was the major factor that affected genetic diversity. AMOVA showed that significant genetic differences were detected between the three groups defined as *WI* conditions, with the variance among groups being 8.71% (*p *=* *.005) (Table 6).

### 3.4 Correlation between soil factor variation and genetic variation

**Figure 3:**
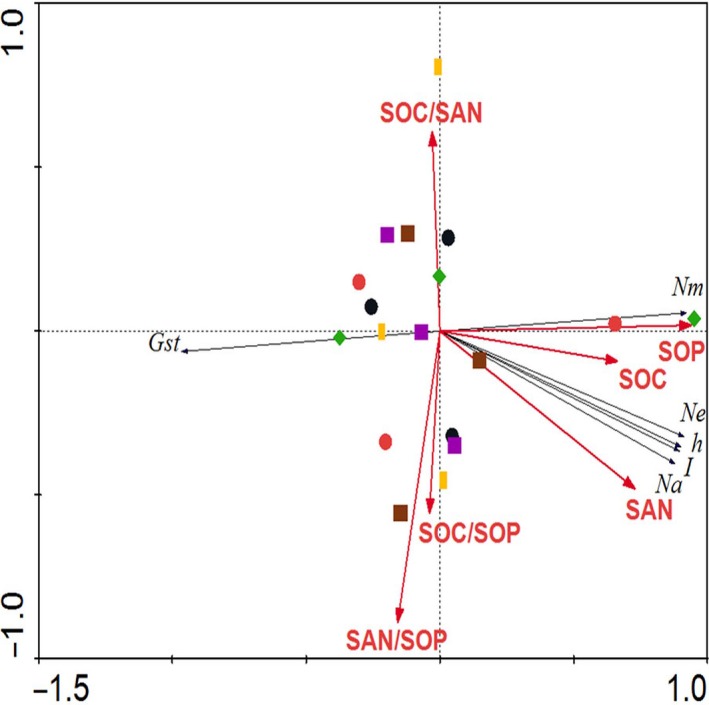
Correlation coefficients between genetic diversity of 20 *Caragana microphylla* populations and soil factors

The genetic diversities of the 20 *C. microphylla* populations from Horqin Sandy Land were influenced by the following soil factors: SOC, SAN, SOP, SOC/SAN, SOC/SOP, and SAN/SOP (Figure 3). Correlation analyses revealed that there were positive correlations between genetic diversity of *C. microphylla* populations and certain soil factors (SOC, SAN, and SOP) (Figure 3). RDA showed that the six soil environmental variables (SOC, SAN, SOP, SOC/SAN, SOC/SOP, and SAN/SOP) together explained 88.6% of the total variation in the data, with axes 1 and 2 explaining 86.1% and 2.5% of the total variation, respectively (Figure 3). Of the six soil environmental variables, only SOP was significant according to the Monte Carlo permutation test (*p* = .001), whereas those of the remaining variables were not (in all cases *p* > .05). Of the total 88.6% variation explained by RDA, 76.1% was explained by SOP and the rest (12.5%) by variables that were not significant according to the Monte Carlo permutation test.

Using RDA, we assessed the relative contribution of the measured soil environmental factors in determining genetic diversity indices of *C. microphylla* populations. RDA revealed that the six environmental factors (SOC, SAN, SOP, SOC/SAN, SOC/SOP, and SAN/SOP) together explained 88.6% of the total variation in the genetic diversity indices of *C. microphylla* populations from Horqin Sandy Land. This result indicates that some other soil factors that were not considered in this study also contribute to the unexplained variation. SOP was the major factor that affects genetic diversity. AMOVA showed that significant genetic differences were detected between the three groups defined as SOP conditions, with the variance among groups being 8.65% (*p *=* *.004) (Table 6).

## 4 Discussion

### 4.1 Genetic variation

Genetic diversity of a species is dynamic and shaped by processes that act on widely different spatial and temporal scales. Assessing genetic variation is thus an important component of plant conservation and ecological restoration. In our study, *C. microphylla* generally exhibited high levels of genetic diversity. At the species level, the value of *h* estimated in our study for *C. microphylla*, 0.3365, is higher than that reported from RAPD markers for *Stipa grandis* from Inner Mongolia (0.2305) (Zhao, Gao, Wang, & Ren, 2008). In *C. microphylla*, we calculated a value of 0.5041 for *I*; this is higher than the mean *I* value for outcrossing species produced by Bussell (1999). Compared with previous study, all values of genetic diversity indices estimated in *C. microphylla* in Horqin Sandy Land are higher than those reported in northern China based on ISSR analysis (Huang et al., 2016). These differences came from scale difference, because of Horqin Sandy Land was the main natural distribution area of *C. Microphylla*. On the small scale, the population quantity and the single population size are larger, which makes the gene flow more frequently. *C. microphylla* is the important sand‐fixation plant in northern China, and there are cultivated populations in natural distribution areas. The genetic diversity indices estimated in this study are higher than plantation populations (Huang et al., 2013). Several reports have indicated that the genetic diversity of natural populations was higher compared with other population type (Chen, Gao, Zhu, & Zhao, 2009; Xue, Liu, & Liu, 1998). But other authors (Hamrick & Godt, 1990; Nybom & Bartish, 2000) noted that levels of genetic variation are strongly dependent on plant life form, geographic range, pollen dispersal mechanisms, and natural selection. *Caragana microphylla* are long‐lived, perennial, undergo wind pollination, have seed‐based reproduction, and have a broad ecological amplitude. Based on the findings of previous reports, this combination of traits should enable this species to achieve high genetic diversity (Babbel & Selander, 1974; Ge, Wang, Hong, Zhang, & Zu, 1999; Pearse, Crandall, & Beyond, 2004). *Caragana microphylla* does exhibit high levels of genetic diversity, and this characteristic may have contributed to it being a dominant species in northern China.

### 4.2 Correlation between genetic variation and environmental gradients

Along the habitat gradient, *C. microphylla* populations from fixed‐state sand dunes (fixed sand dune and lowlands between fixed sand dunes) had greater genetic diversity than those from mobile‐state sand dunes (mobile sand dunes and lowlands between mobile sand dunes), based on a variety of genetic diversity indices. These results showed that habitat environment change affected genetic diversity of *C. microphylla* populations, which is consistent with the results about *Artemisia halodendron* (Huang et al., 2011). These genetic differences between the two types of habitats exhibited that species adapted to the process of desertification land restoration in Horqin Sandy Land, northeast China (Zhao et al., 2000, 2003). This fact might be due to improved soil environments and microclimatic conditions in fixed‐state sand dunes, and individual shrubs were more concentrated than populations from mobile‐state sand dunes, leading to pollen exchange.

In our study, population genetic diversity of *C. microphylla* was not well correlated with the temperature gradient in Horqin Sandy Land, northeast China. Populations from the high‐temperature region had lower genetic diversity than those from medium‐ and low‐temperature regions. However, these results were not consistent with the results of *C. microphylla* at relatively smaller geographic distances in Horqin Sandy Land (Huang et al., 2015), which showed that increased temperature, geographical distance, and population size affected genetic diversity of *C. microphylla* populations. Our data show that *C. microphylla* population genetic diversity is related to humidity gradients. Populations from the high‐humidity region had higher genetic diversity than those from the medium‐ and low‐humidity regions region. These results showed that genetic diversity of *C. microphylla* populations was reflected by humidity conditions at a small scale in northern China. These results indicate that desert plants would be able to adapt to an increase in humidity; therefore, genetic differentiation of species would be expected to increase as a result of adaptation (Wang et al., 2009).

### 4.3 Correlation between environmental and genetic data

Ecological and environmental factors can play roles in shaping genetic diversity patterns (Gaggiotti et al., 2009). With regard to the whole ecosystem, relationships between genomes and environmental factors (e.g., temperature factors, humidity factors, and soil factors) are considered important components of ecological research (Li & Peng, 2001). In our study, correlation analysis revealed a positive association between genetic diversity of *C. microphylla* and most environmental factors (*WI*, AP, *S*, SOC, SAN, and SOP). However, there were negative associations between genetic diversity and a couple of environmental factors (AMT and *CI*). This result reflected that genetic diversity of *C. microphylla* was restrained by AMT and *CI*. This result is not consistent with the previously reported relationship between ISSR diversity and AMT in *A. halodendron* from Horqin Sandy Land (Huang et al. 2014). Our study revealed that *WI* and SOP were the major environmental factors that affected genetic diversity indices of the 20 studied *C. microphylla* populations, which indicates that genetic variation in *C. microphylla* primarily depends on a greater than 5°C monthly mean temperature and SOP concentration. This result is not consistent with the previously reported relationship between ISSR diversity and environmental factors in *C. microphylla* from northern China (AP and *CI* were the major environmental factors that affected genetic diversity indices) (Huang et al., 2016). This suggests that AP and *CI* were the major environmental factors that affected genetic diversity indices in *C. microphylla* on the large‐scale field, and *WI* was the major environmental factors on the small‐scale field. Relevant report found that SAN, SOP, and SAN/SOP contents can exert a chemical control over the evolution of species through changing an organism's rate of growth, or an adaptation to each situation of SAN/SOP (Acquisti, Elser, & Kumar, 2009). However, evaluation of other environmental factors that impact plant population genetic diversity requires further research.

## Conflict of Interest

None declared.
